# Supplementary material for: Resonant perovskite solar cells with extended band edge
Source: Nat Commun. 2023 Sep 5;14:5392. doi: 10.1038/s41467-023-41149-1 (PMC10477336; doi:10.1038/s41467-023-41149-1)
Supplement: Supplementary file 1 — Supplementary Information [file 41467_2023_41149_MOESM1_ESM.pdf]

Supplementary Information

Resonant Perovskite Solar Cells with Extended Band Edge

Jiangang Feng[1, 2, †], Xi Wang[1, 2, †], Jia Li[1, 2], Haoming Liang[1, 2], Wen Wen[3], Ezra Alvianto[1, 2], Cheng-Wei Qiu[4], Rui Su[3, 5], Yi Hou[1, 2, *]

[1] Department of Chemical and Biomolecular Engineering, National University of Singapore, Singapore 117585, Singapore

[2] Solar Energy Research Institute of Singapore (SERIS), National University of Singapore, Singapore 117574, Singapore

[3] Division of Physics and Applied Physics, School of Physical and Mathematical Sciences, Nanyang Technological University, Singapore 637371, Singapore

[4] Department of Electrical and Computer Engineering, National University of Singapore, Singapore 117583, Singapore

[5] School of Electrical and Electronic Engineering, Nanyang Technological University, Singapore 639798, Singapore

† These authors contributed equally to this work

*Email: yi.hou@nus.edu.sg

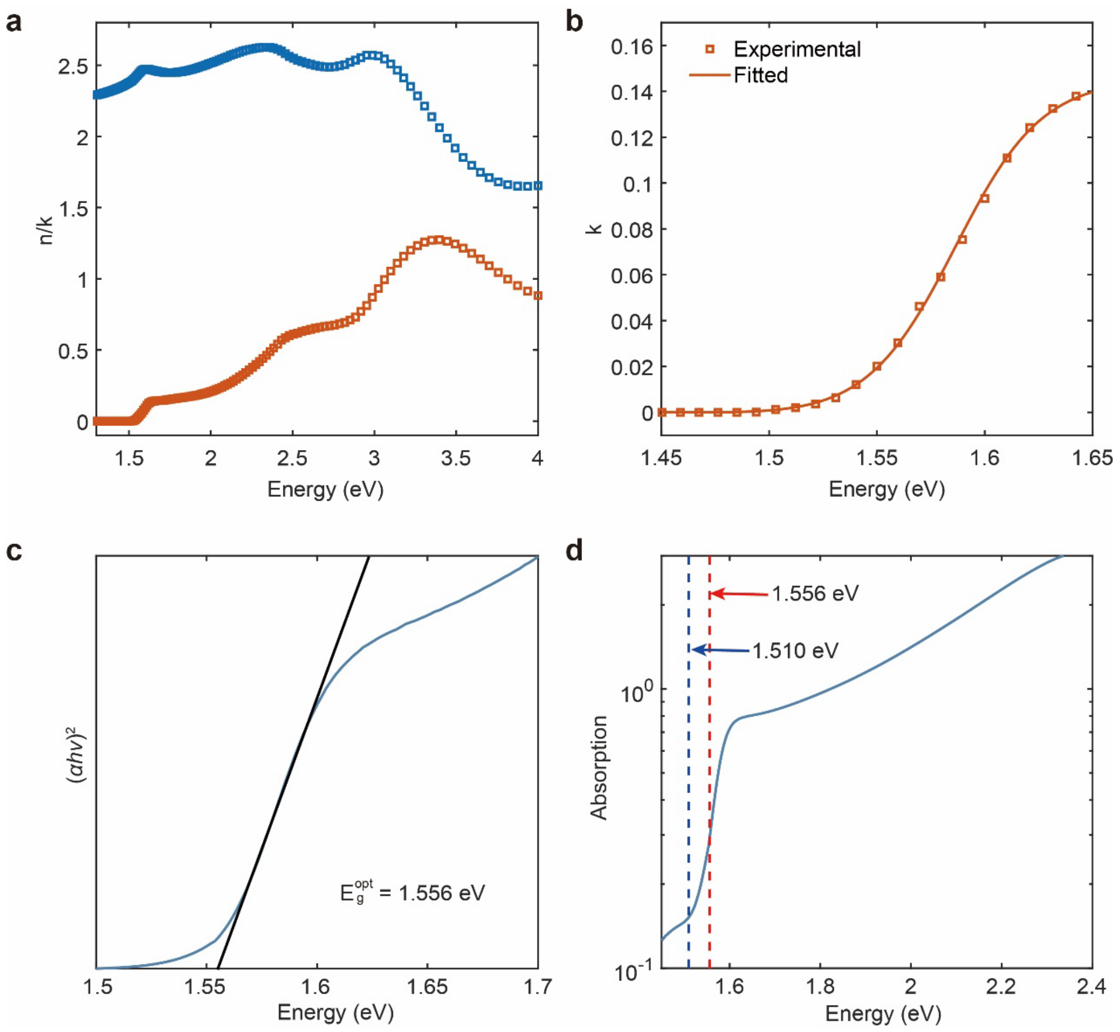

Supplementary Fig. 1 | Optical properties of mixed cation perovskites. **a**, Experimental refractive index *n* and extinction coefficient *k* of perovskites. **b**, Experimental and fitted *k* for the FDTD simulation. **c**, Tauc plot determines an optical bandgap of 1.556 eV. **d**, Absorption spectrum in logarithmic scale, indicating non-zero absorption coefficient below the optical bandgap. Finite absorption coefficient ranging from 1.510 to 1.556 eV allows for narrowing PV bandgap below optical bandgap.

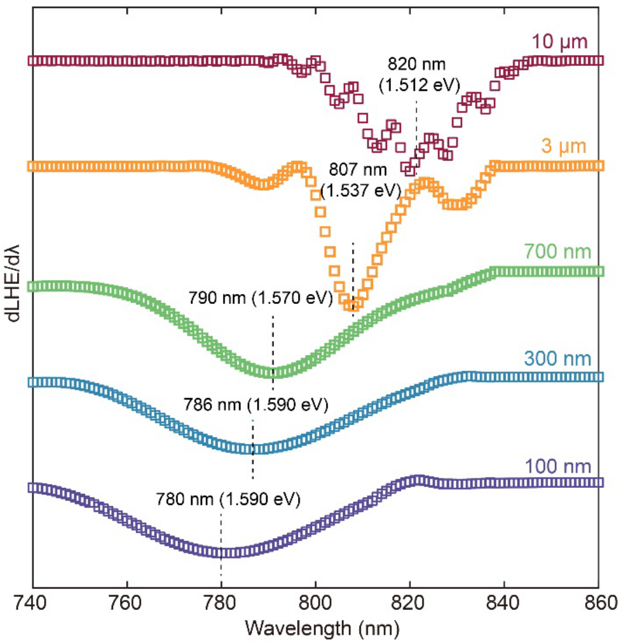

Supplementary Fig. 2 | First derivative of simulated LHE spectra for determining PV bandgap. Simulated dLHE/dλ spectra of single-crystal perovskite solar cells with perovskite thickness ranging from 100 nm to 10 μm.

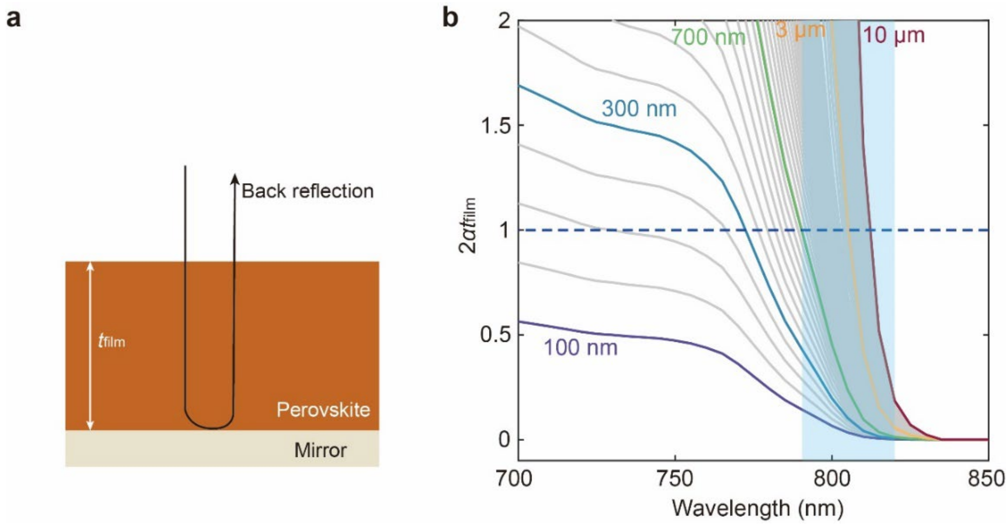

Supplementary Fig. 3 | Target wavelength for PV bandgap narrowing. a, Schematic of double-pass optical absorption in perovskites. **b**, Double-pass perovskite absorption evaluated by $2\alpha t$, where α is absorption coefficient, and t is perovskite thickness.

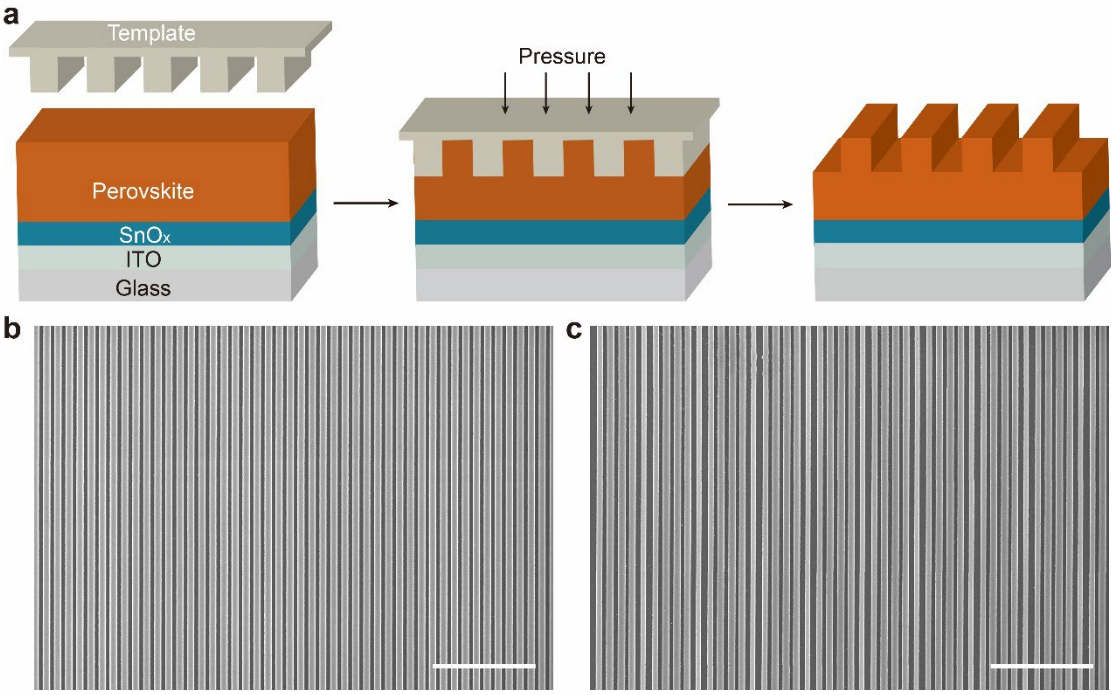

Supplementary Figure 4 | Nanoimprinting for the fabrication of perovskite resonant structures. a, Schematic illustration of nanoimprinting for the fabrication of perovskite resonant structures. A silicon template and a perovskite thin film are combined with an applied pressure, enabling conformal transfer of patterns from template to perovskite. SEM images of silicon templates for the fabrication of (**b**) single-cell, (**c**) supercell gratings. Scale bars, 5 μm.

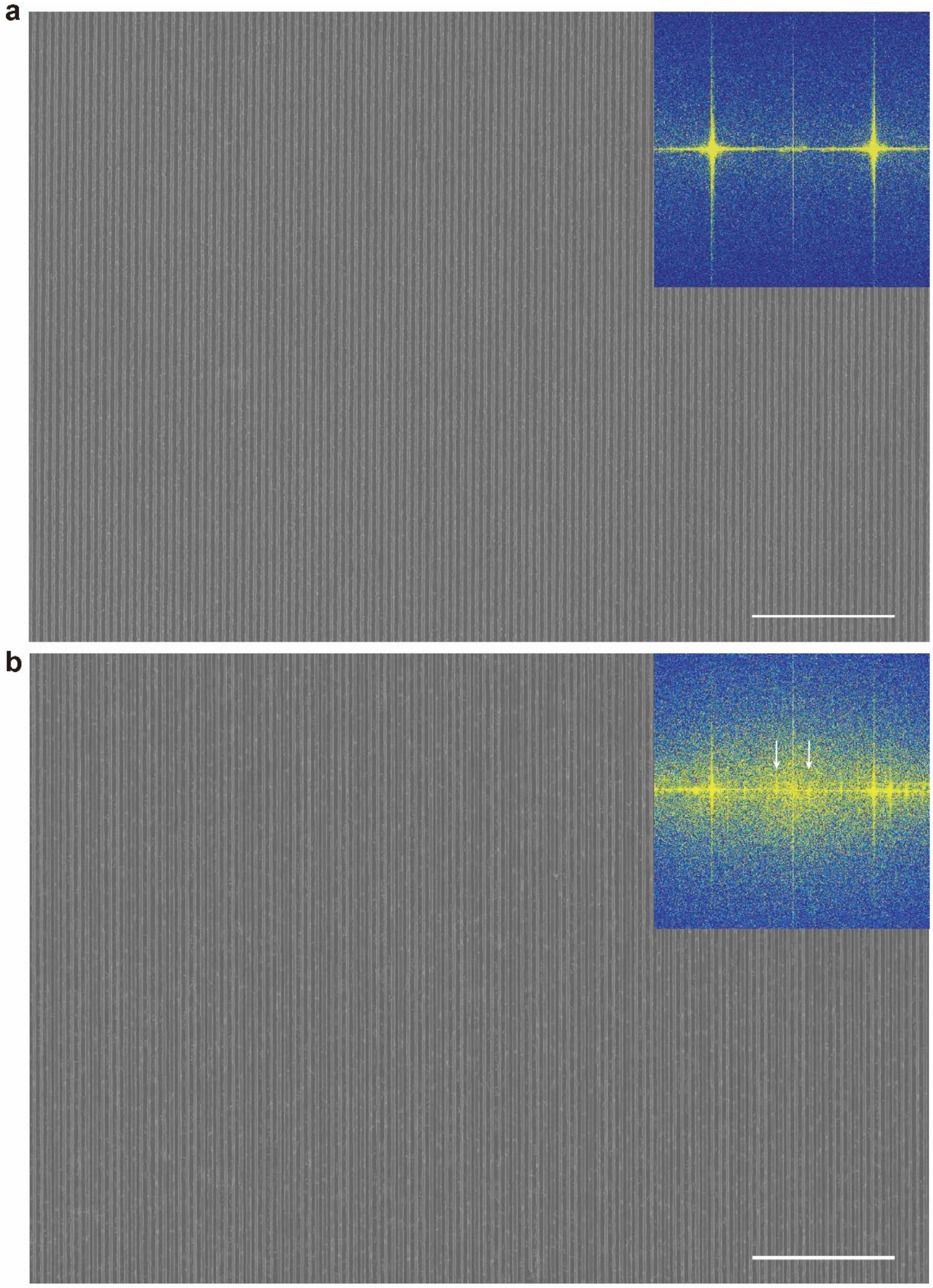

Supplementary Fig. 5 | SEM image of perovskite resonant structures. a, SEM image of a perovskite single-cell grating. Inset shows the FFT pattern extracted from the SEM image. Two pronounced FFT components appear in the periodic direction of the perovskite grating, indicating its long-range homogeneity. **b**, SEM image of a perovskite supercell grating. Inset is the FFT pattern extracted from the SEM image of a supercell grating. FFT components of supercell gratings (labeled by white arrows) indicate the long-range homogeneity. Scale bars, 10 µm.

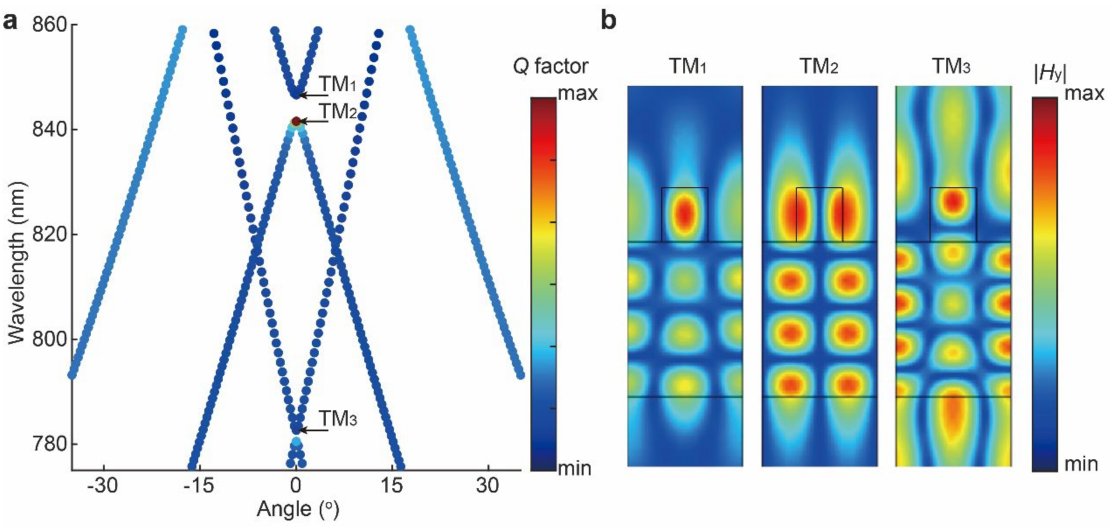

Supplementary Fig. 6 | Simulated photonic bandstructure of a single-cell grating. **a,** Photonic bandstructure of a low-contrast single-cell grating with p = 535 nm, t_g = 250 nm and t_s = 680 nm. The band structure is calculated under TM polarization. **b**, Magnetic-field $|H_y|$ profile of TM modes.

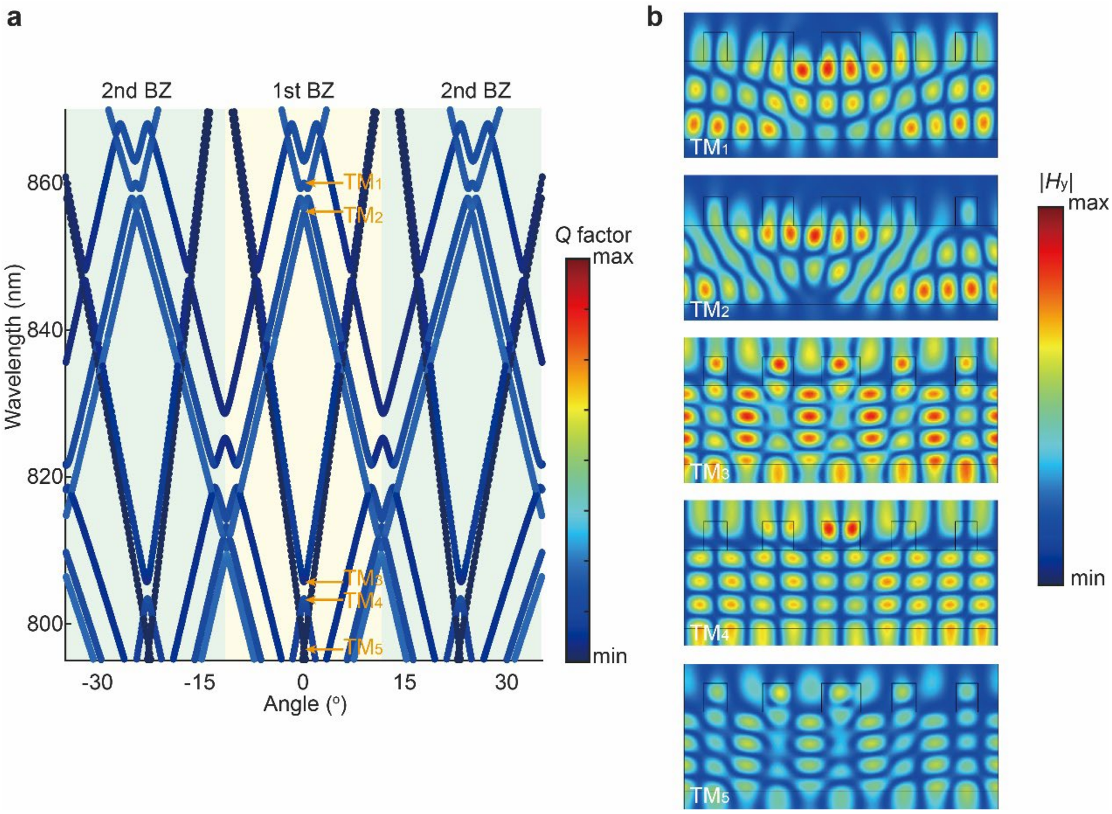

Supplementary Fig. 7 | Simulated photonic bandstructure of a supercell grating. **a,** Photonic bandstructure of a supercell grating with p = 535 nm, t_g = 250 nm, t_w = 680 nm. The bandstructure is calculated by constructing a layer of perovskite grating on glass substrate. **b**, Magnet-field profile of TM modes.

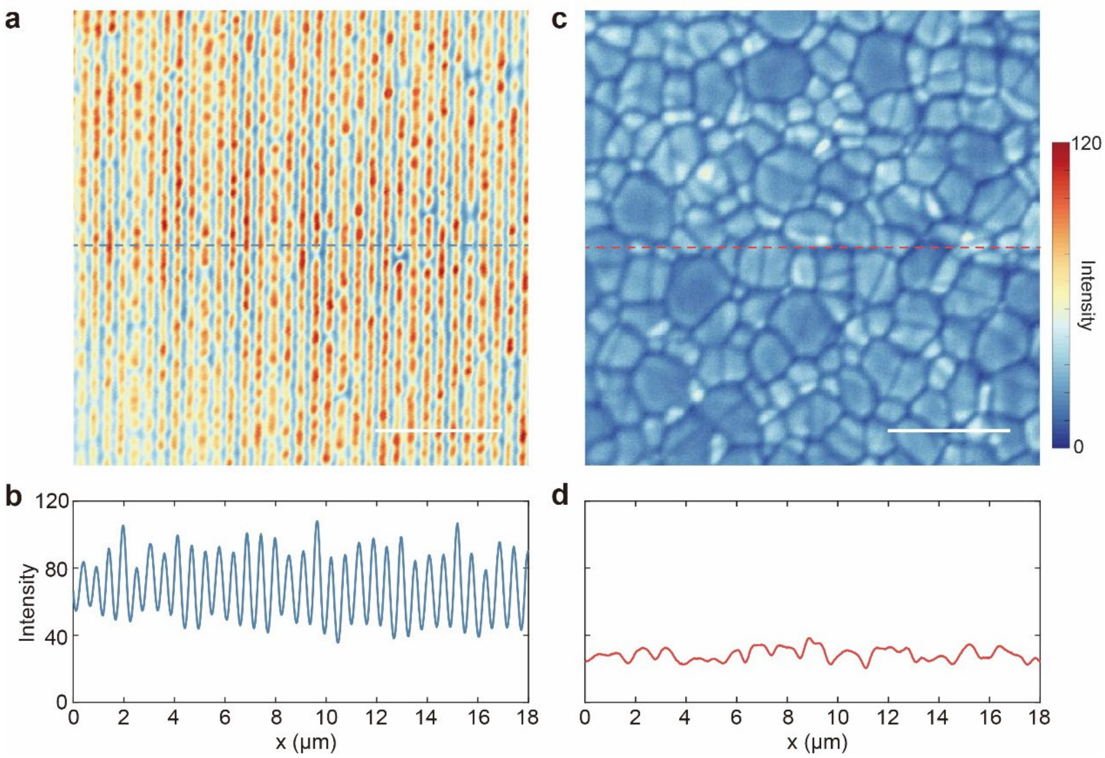

Supplementary Fig. 8 | Linecuts of confocal PL mapping. **a**, **b**, Confocal PL mapping (**a**), and linecut of PL map (**b**) of a perovskite grating. **c**, **d**, Confocal PL mapping (**c**), and linecut of PL map (**d**) of a perovskite thin film. The periodicity in the confocal PL imaging is attributed to the principle of confocal PL imaging, in which a pinhole is applied to eliminate out-of-focus light signals. The grating ridge was chosen as the focal plane to enable the spatial resolution of PL imaging, thus leading to a brighter PL signal from the ridges compared to the valleys.

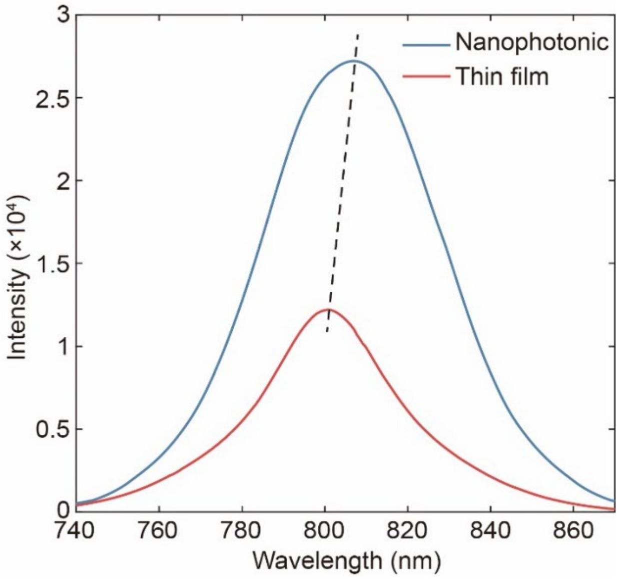

Supplementary Fig. 9 | PL spectra of perovskite resonant structures and thin films. Dashed line is a guide to the eye.

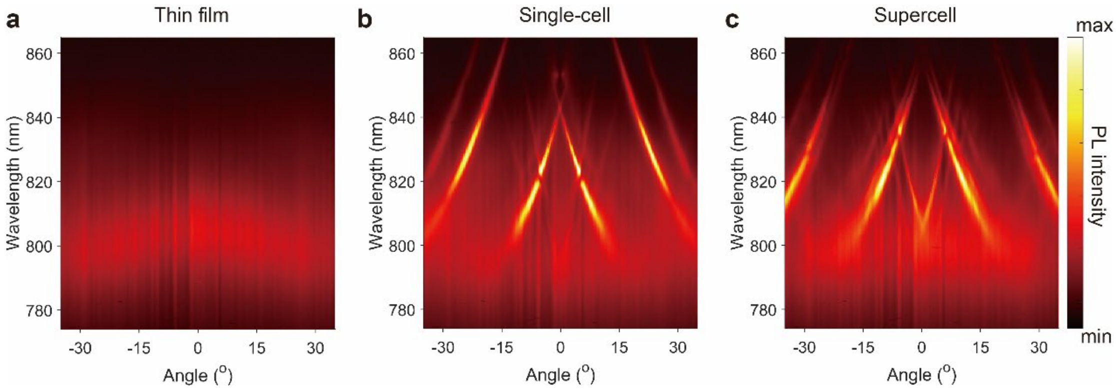

Supplementary Fig. 10 | Angle-resolved PL spectra. a-c, Angle-resolved PL spectra of perovskite thin film (**a**), single-cell (**b**), and supercell (**c**) gratings. All samples were excited with a 457 nm continuous-wave laser at the same power.

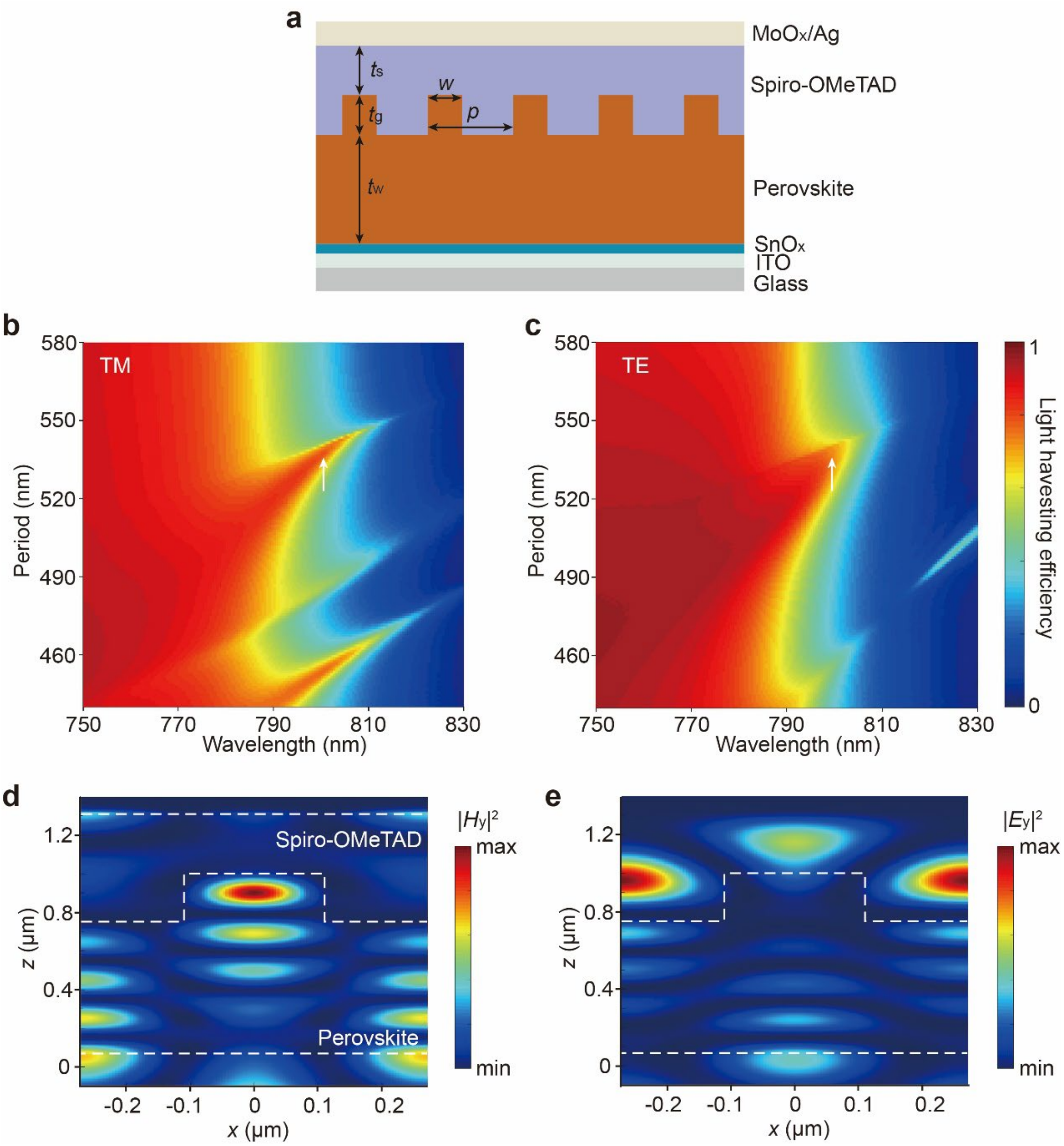

Supplementary Fig. 11 | Simulation of dispersion diagram and resonance modes in a solar cell with a single-cell grating. a, Device configuration of a resonant solar cell based on a single-cell grating. The simulations are performed on a solar cell structure of glass/ITO/SnO_2/perovskite/Spiro-OMeTAD/MoO_x/Ag. The parameters are t_s = 310 nm, t_g = 250 nm, and t_w = 680 nm. The duty ratio is given by $DC = w/p$, which is kept as 0.4 during the sweep of grating period. Simulated LHE spectra as a function of grating period under **b**, transverse magnetic (TM), **c**, transverse electric (TE) polarization. **d**, Cross-sectional magnetic-field profile of TM mode and **e**, electric-field profile of TE mode in a solar cell with a single-cell grating.

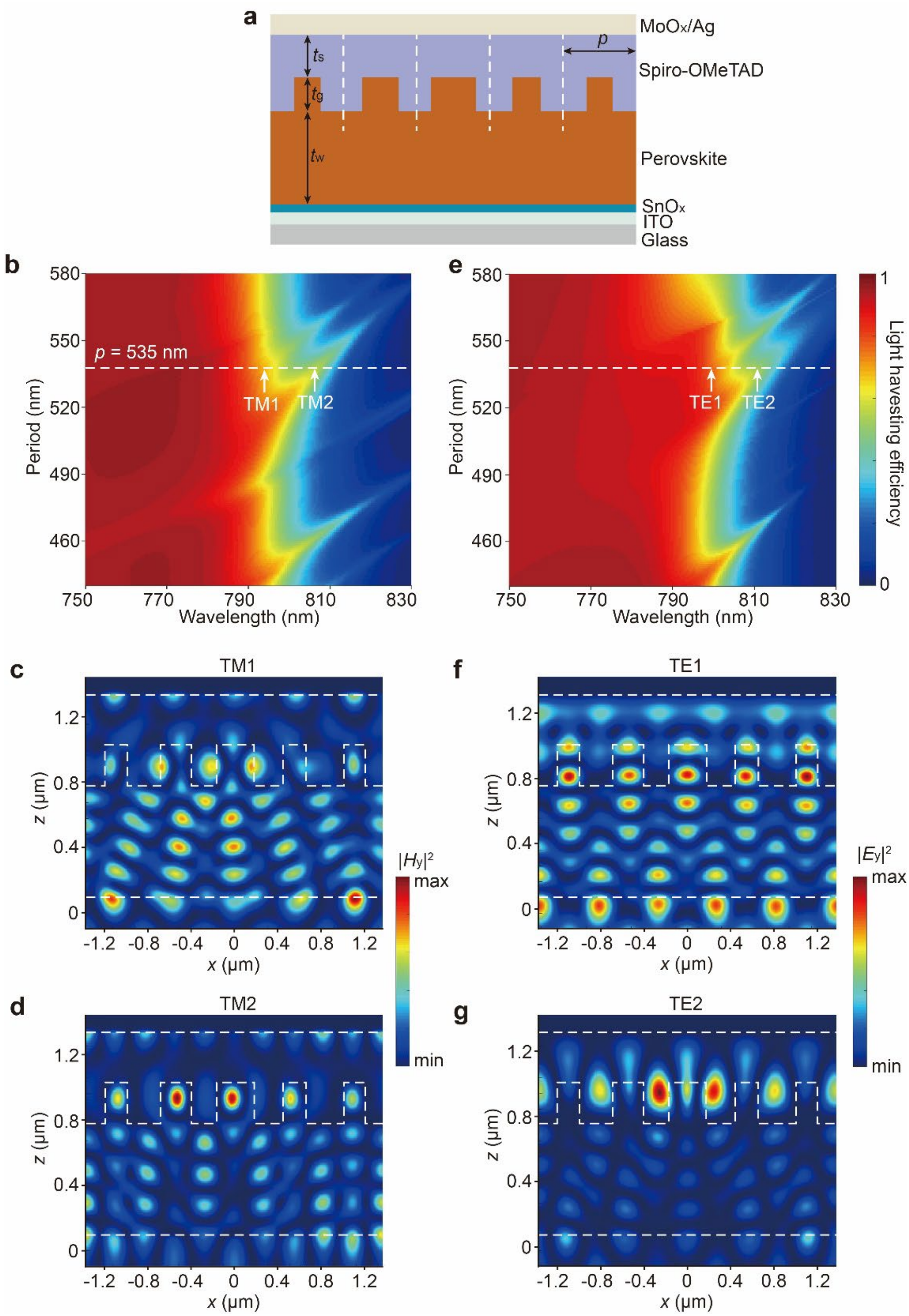

Supplementary Fig. 12 | Simulation of dispersion in resonant solar cells with a supercell grating. a, Device configuration of a solar cell based on a supercell grating. The parameters are t_s = 310 nm, t_g = 250 nm, and t_w = 680 nm. Each unit cell of a supercell grating contains five ridges with duty ratios of 0.37, 0.50, 0.62, 0.39, and 0.35. **b**, Dispersion of LHE as a function of period *p* and wavelength under TM polarization. Magnetic-field profile of **c**, TM1 mode, **d**, TM2 mode. **e**, Dispersion of LHE as a function of period and wavelength under TE polarization. Electric-field profile of **f**, TE1 mode, **g**, TE2 mode.

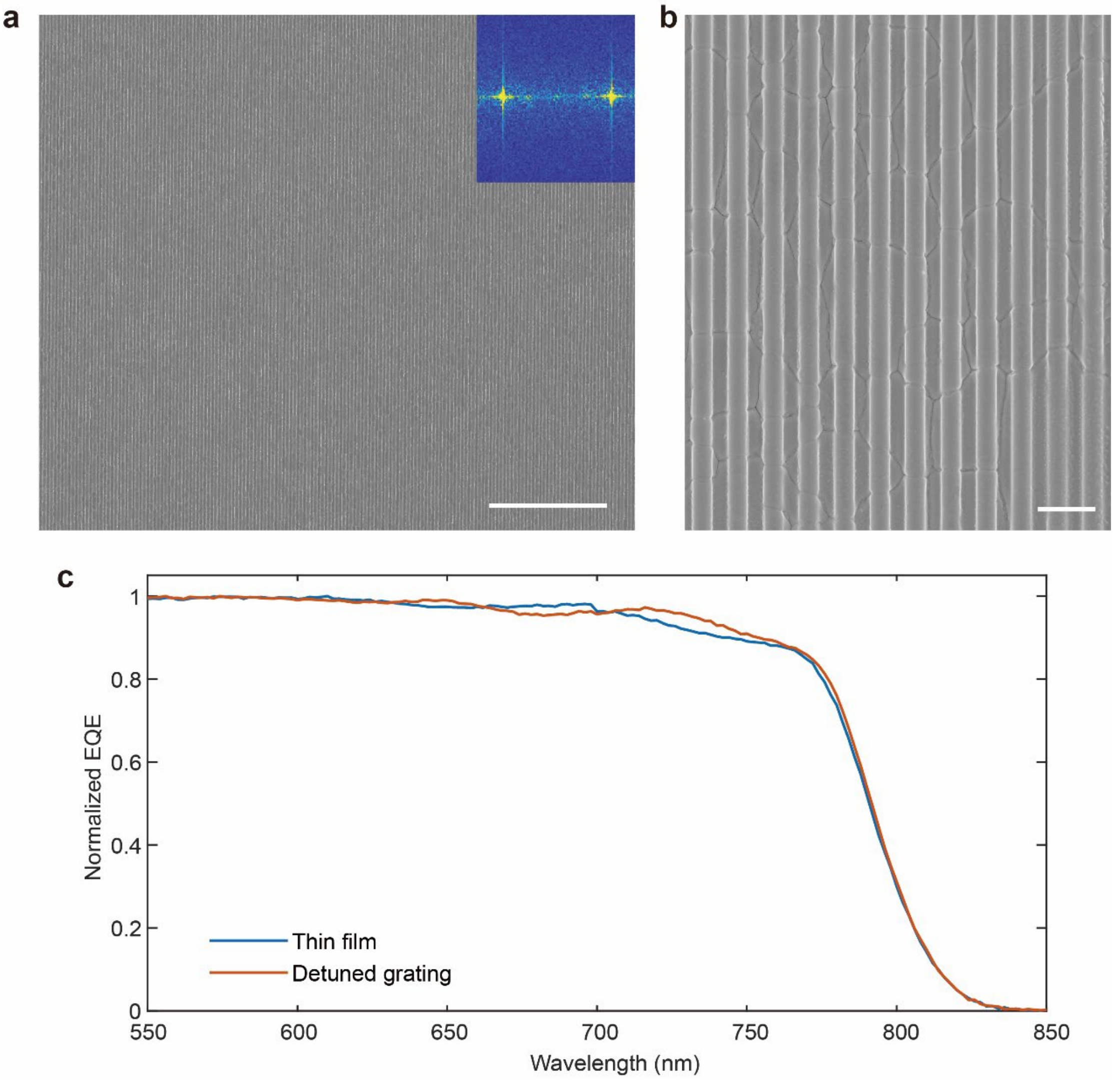

Supplementary Fig. 13 | Solar cells with an off-resonance grating. **a**, Low-magnification SEM image of a grating with a period p = 615 nm, a ridge width w = 350 nm, and a height t_g = 250 nm. Inset shows 2D FFT pattern extracted from the SEM image. **b**, Zoom-in SEM image of an off-resonance grating. **c**, Normalized EQE solar cells based on thin film and off-resonance grating. Scale bars, **a**, 10 μm, **b**, 1 μm.

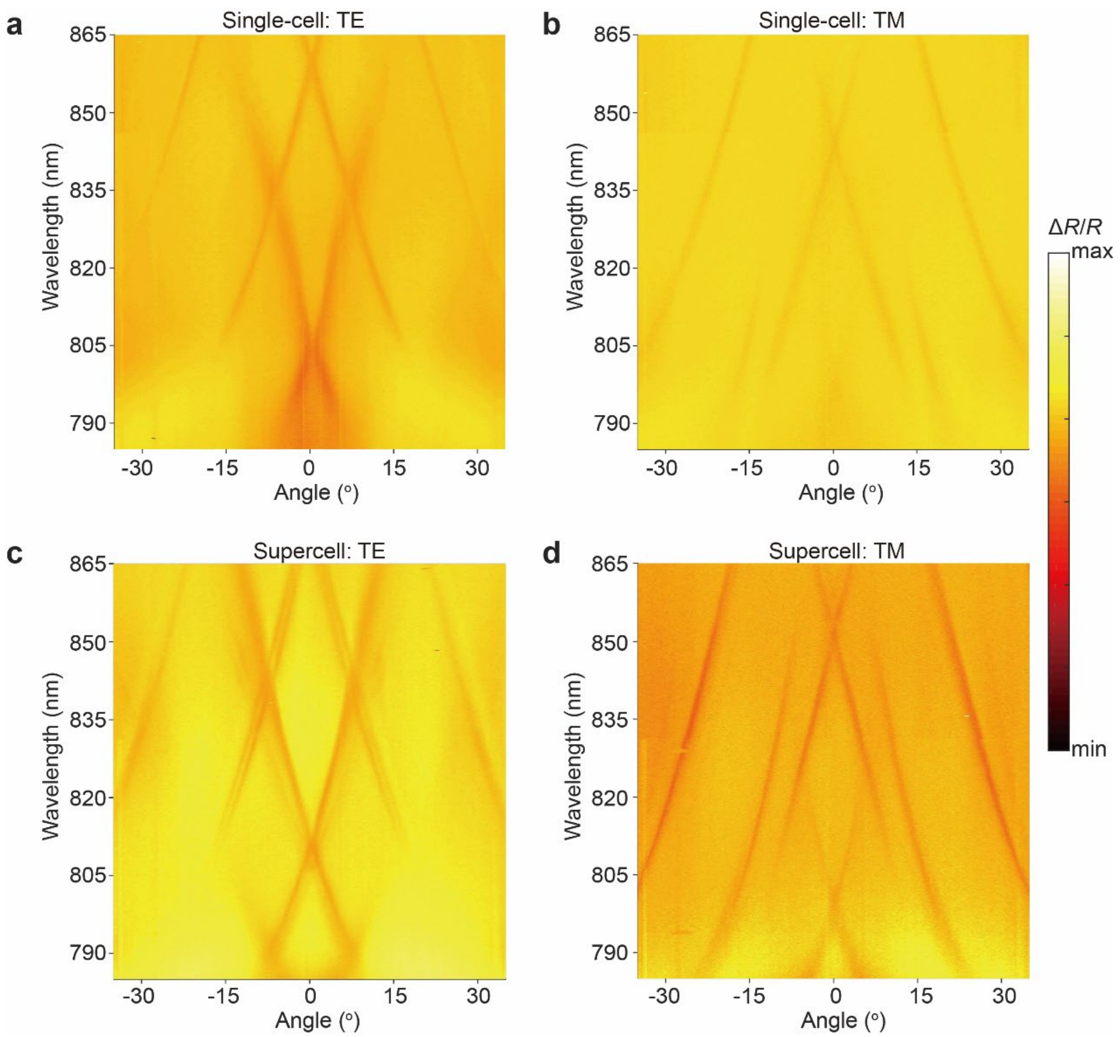

Supplementary Fig. 14 | Angle-resolved reflectance spectra of resonant solar cells. Angle-resolved reflectance spectra of a solar cell with **a**, **b**, a single-cell grating and **c**, **d**, a supercell grating under TE and TM polarizations. The angle-resolved reflectance spectra of resonant and planar solar cells are measured and the $\Delta R/R$ is calculated by $\Delta R/R = (R_{np} - R_{planar})/ R_{planar}$, where R_{np} and R_{planar} denote reflectance of resonant and thin-film solar cells, respectively.

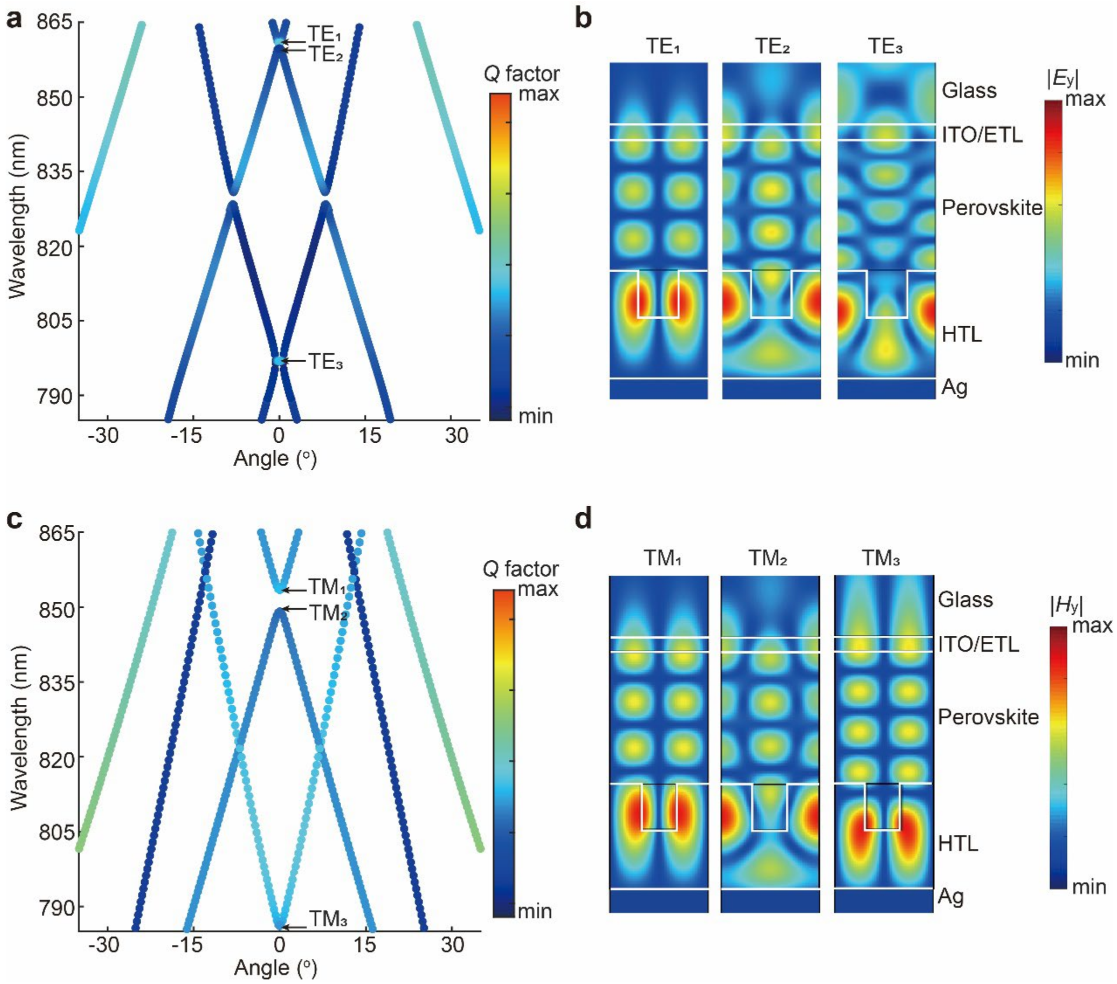

Supplementary Fig. 15 | Simulated photonic bandstructure and resonance modes of a solar cell with a single-cell grating. Photonic bandstructure of a resonant solar cell under **a**, TE polarization, and **c**, TM polarization. A full solar cell with glass/ITO/ETL/perovskite/HTL/Ag is considered for bandstructure calculations. Geometric parameters include thickness of ITO t_{ITO} = 80 nm, thickness of SnO_x t_{SnO} = 20 nm, t_w = 680 nm, t_g = 250 nm, t_s = 310 nm, p = 535 nm, and w = 214 nm. Electric/magnetic field profile of **b**, TE modes, **d**, TM modes.

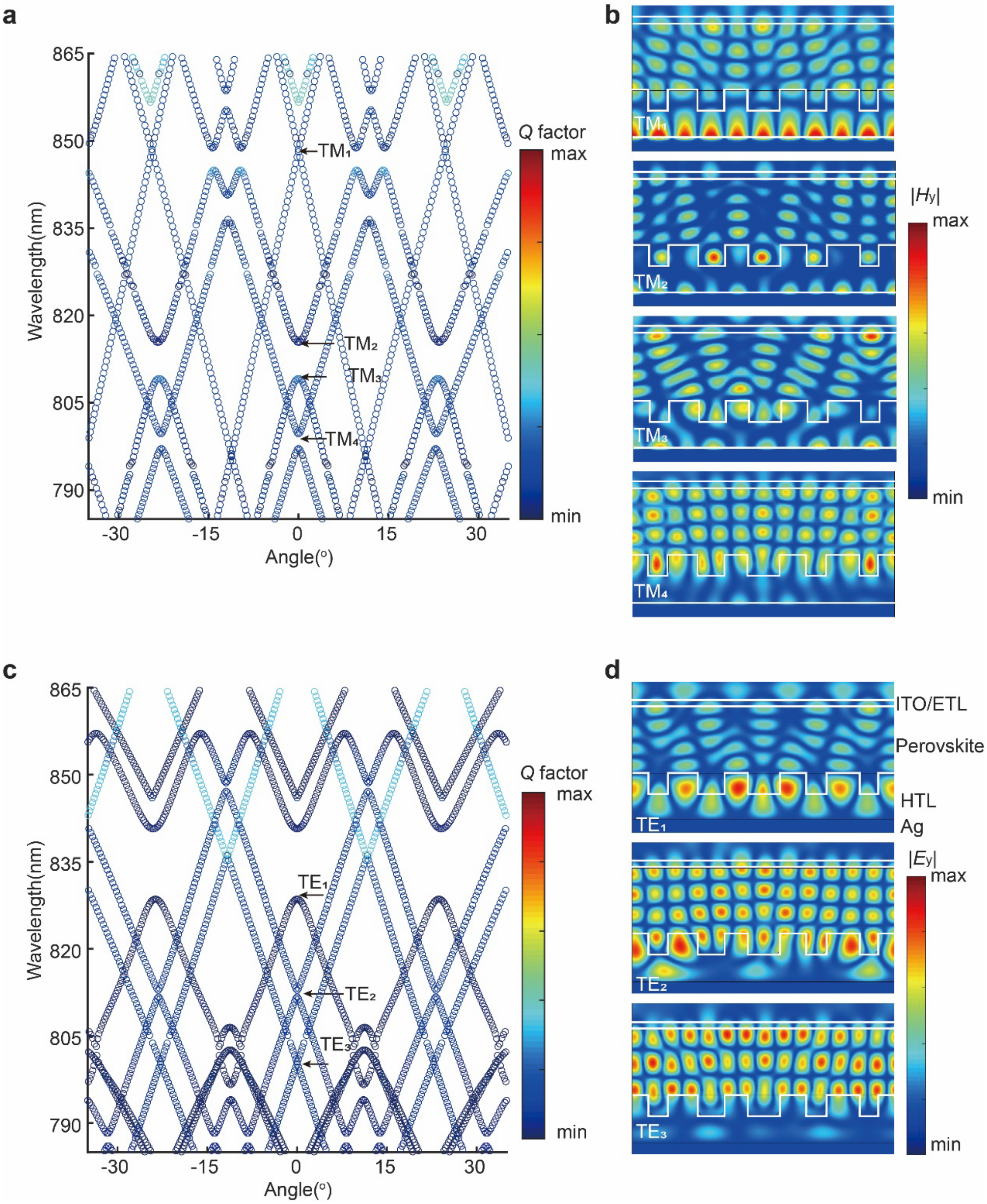

Supplementary Fig. 16 | Simulated photonic bandstructure of a solar cell based on a supercell grating. Photonic bandstructure of a supercell-grating solar cell under **a,** TM, and **c**, TE polarization. All geometric parameters are consistent with those used in FDTD simulation and experiments. Magnetic/electric field profiles of **b**, TM, **d**, TE modes.

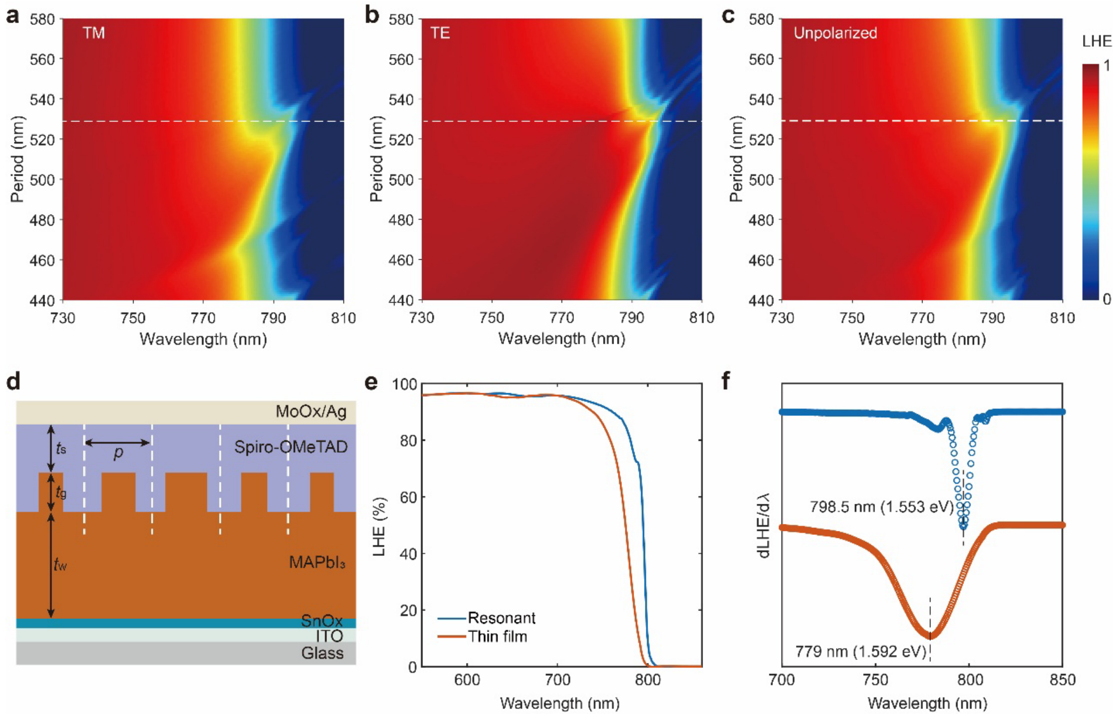

Supplementary Fig. 17 | Simulations of a resonant perovskite solar cell based on $MAPbI_3$. a-c, FDTD simulations of LHE spectra as a function of grating period under TM (**a**), TE (**b**), and unpolarized (**c**) light. **d**, Device configuration for the optical simulation of the resonant perovskite solar cell utilizing a supercell grating. The parameters are t_s = 280 nm, t_g = 250 nm, and t_w = 660 nm. The refractive index of $MAPbI_3$ is extracted from Ref 1. **e**, Simulated LHE spectra of thin-film and resonant perovskite solar cells. The period of the supercell grating is 528 nm. **f**, Simulated dLHE/dλ spectra of thin-film and resonant perovskite solar cells, illustrating the extension of the band edge after introducing optical resonances.

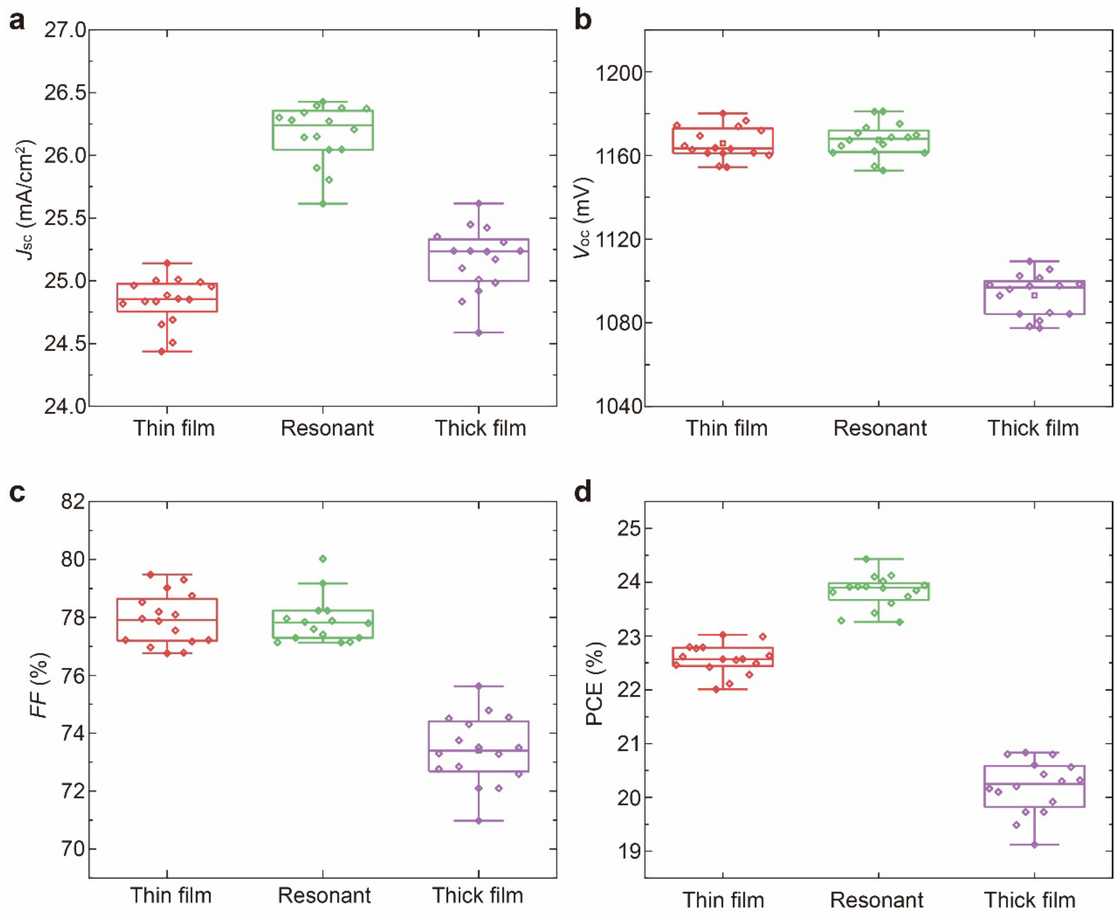

Supplementary Fig. 18 | Statistics of device performances. a-d, Statistics of J_{sc} (**a**), V_{oc} (**b**), *FF* (**c**), and PCE (**d**) based on 16 devices for each type of solar cells.

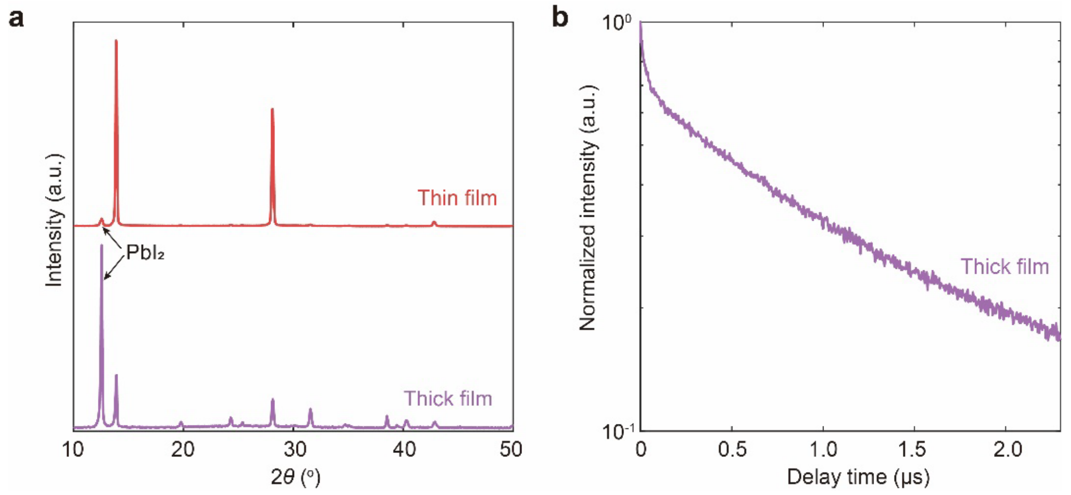

Supplementary Fig. 19 | XRD and TRPL of perovskite thick film. a, XRD diagrams of perovskite thin film and thick film. **b**, TRPL of perovskite thick film.

Supplementary Table 1 | Comparison of light-management strategies in solar cells. The subscript *a* represents EQE-integrated J_{sc}, and *b* represents J_{sc} extracted from *IV* scan.

Light-management strategy	Optics	J_{sc} (mA/cm^2)	Efficiency	Ref
**Folded guided mode resonance**	**Wave optics**	**26.0_a 26.3_b**	**24.4%**	**Our work**
**Texture**	Ray optics	22.0_a 22.3_b	16.3%	2
**Texture**	Ray optics	21.3_b	17.7%	3
**Texture**	Ray optics	23.6_b	19.8%	4
**Texture**	Ray optics	21.7_b	17.1%	5
**Texture**	Ray optics	22.7_a 23.5_b	21.8%	6
**Texture**	Ray optics	23.1_a 24.4_b	22.2%	7
**Texture**	Ray optics	21.9_b	16.3%	8
**Diffraction**	Ray optics	23.1_b	19.7%	9
**Diffraction**	Ray optics	24.2_b	19.6%	10
**Diffraction**	Ray optics	23.7_b	21.8%	11

Supplementary Table 2 | State-of-the-art perovskite solar cells with different perovskite compositions. Subscript *a* and *b* denote PCE with and without certification, respectively.

Year	Perovskites	PCE (%)	Voc (V)	FF (%)	J_{sc} (mA/cm^2)	Ref
2023	$FAPbI_3$	25.73_a	1.179	84.6	25.80	12
2022	$FAPbI_3$	25.6_a	1.182	82.7	26.30	13
2022	$MAPbI_3$	22.52_b	1.20	79.52	23.60	14
2022	$CsPbI_3$	21.0_b	1.20	84.1	20.86	15
2021	$FA_{0.6}MA_{0.4}PbI_3$ single crystal	22.8_b	1.1	0.79	26.2	16
2021	$FAPbI_3$	25.5_a	1.189	83.2	25.74	17
2021	$FAPbI_3$	25.4_a	1.177	81.5	26.28	18
2021	$(FAPbI_3)_{0.975}(MAPbBr_3)_{0.025}$	25.2_a	1.181	84.8	25.14	19
2020	$FAPbI_3$	24.64_a	1.181	79.6	26.18	20

Supplementary Reference

1 Manzoor, S. *et al.* Optical modeling of wide-bandgap perovskite and perovskite/silicon tandem solar cells using complex refractive indices for arbitrary-bandgap perovskite absorbers. *Opt. Express* **26**, 27441-27460 (2018).

2 Pascoe, A. R. *et al.* Enhancing the optoelectronic performance of perovskite solar cells via a textured CH3NH3PbI3 morphology. *Adv. Funct. Mater.* **26**, 1278-1285 (2016).

3 Dudem, B., Heo, J. H., Leem, J. W., Yu, J. S. & Im, S. H. CH3NH3PbI3 planar perovskite solar cells with antireflection and self-cleaning function layers. *J. Mater. Chem. A* **4**, 7573-7579 (2016).

4 Zhang, H., Kramarenko, M., Osmond, J., Toudert, J. & Martorell, J. Natural Random Nanotexturing of the Au Interface for Light Backscattering Enhanced Performance in Perovskite Solar Cells. *ACS Photon.* **5**, 2243-2250 (2018).

5 Jošt, M. *et al.* Efficient Light Management by Textured Nanoimprinted Layers for Perovskite Solar Cells. *ACS Photon.* **4**, 1232-1239 (2017).

6 Wang, F. *et al.* Coordinating light management and advance metal nitride interlayer enables MAPbI3 solar cells with >21.8% efficiency. *Nano Energy* **92**, 106765 (2022).

7 Tavakoli, M. M. *et al.* Ambient stable and efficient monolithic tandem perovskite/PbS quantum dots solar cells via surface passivation and light management strategies. *Adv. Funct. Mater.* **31**, 2010623 (2021).

8 Wei, J. *et al.* Enhanced Light Harvesting in Perovskite Solar Cells by a Bioinspired Nanostructured Back Electrode. *Adv. Energy Mater.* **7**, 1700492 (2017).

9 Wang, Y. *et al.* Diffraction-Grated Perovskite Induced Highly Efficient Solar Cells through Nanophotonic Light Trapping. *Adv. Energy Mater.* **8**, 1702960 (2018).

10 Deng, K., Liu, Z., Wang, M. & Li, L. Nanoimprinted Grating-Embedded Perovskite Solar Cells with Improved Light Management. *Adv. Funct. Mater.* **29**, 1900830 (2019).

11 Wang, Y. *et al.* Colorful efficient moiré-perovskite solar cells. *Adv. Mater.* **33**, 2008091 (2021).

12 Park, J. *et al.* Controlled growth of perovskite layers with volatile alkylammonium chlorides. *Nature* **616**, 724-730 (2023).

13 Zhao, Y. *et al.* Inactive (PbI2)2RbCl stabilizes perovskite films for efficient solar cells. *Science* **377**, 531-534 (2022).

14 Zhuang, X. *et al.* Synergistic Effects of Multifunctional Lanthanides Doped CsPbBrCl2 Quantum Dots for Efficient and Stable MAPbI3 Perovskite Solar Cells. *Adv. Funct. Mater.* **32**, 2110346 (2022).

15 Tan, S. *et al.* Temperature-Reliable Low-Dimensional Perovskites Passivated Black-Phase CsPbI3 toward Stable and Efficient Photovoltaics. *Angew. Chem. Int. Ed.* **61**, e202201300 (2022).

16 Alsalloum, A. Y. *et al.* 22.8%-Efficient single-crystal mixed-cation inverted perovskite solar cells with a near-optimal bandgap. *Energy Environ. Sci.* **14**, 2263-2268 (2021).
17 Min, H. *et al.* Perovskite solar cells with atomically coherent interlayers on SnO2 electrodes. *Nature* **598**, 444-450 (2021).
18 Kim, M. *et al.* Conformal quantum dot-SnO2 layers as electron transporters for efficient perovskite solar cells. *Science* **375**, 302-306 (2022).
19 Yoo, J. J. *et al.* Efficient perovskite solar cells via improved carrier management. *Nature* **590**, 587-593 (2021).
20 Jeong, M. *et al.* Stable perovskite solar cells with efficiency exceeding 24.8% and 0.3-V voltage loss. *Science* **369**, 1615-1620 (2020).
