# Supplementary material for: Resonant perovskite solar cells with extended band edge
Source: Nat Commun. 2023 Sep 5;14:5392. doi: 10.1038/s41467-023-41149-1 (PMC10477336; doi:10.1038/s41467-023-41149-1)
Supplement: Supplementary file 3 — Reporting Summary [file 41467_2023_41149_MOESM3_ESM.pdf]

nature research

Corresponding author(s): Yi Hou

Solar Cells Reporting Summary

Nature Research wishes to improve the reproducibility of the work that we publish. This form is intended for publication with all accepted papers reporting the characterization of photovoltaic devices and provides structure for consistency and transparency in reporting. Some list items might not apply to an individual manuscript, but all fields must be completed for clarity.

For further information on Nature Research policies, including our data availability policy, see Authors & Referees.

Experimental design

Please check: are the following details reported in the manuscript?

1. Dimensions

Item	Yes	No	Details
Area of the tested solar cells	☒	☐	The area of tested solar cells is 0.055 cm2.
Method used to determine the device area	☒	☐	Metal aperture mask (black color)

2. Current-voltage characterization

Item	Yes	No	Details
Current density-voltage (J-V) plots in both forward and backward direction	☐	☒	*Explain why this information is not reported/not relevant.*
Voltage scan conditions<br>*For instance: scan direction, speed, dwell times*	☒	☐	See details in Method.
Test environment<br>*For instance: characterization temperature, in air or in glove box*	☒	☐	See details in Method.
Protocol for preconditioning of the device before its characterization	☐	☒	No preconditioning condition is used.
Stability of the J-V characteristic<br>*Verified with time evolution of the maximum power point or with the photocurrent at maximum power point; see ref. 7 for details.*	☐	☒	Stability is not related to our band edge extending concept.

3. Hysteresis or any other unusual behaviour

Item	Yes	No	Details
Description of the unusual behaviour observed during the characterization	☐	☒	Negligible hysteresis was observed.
Related experimental data	☐	☒	Hysteresis is not relevant to our band edge extending concept.

4. Efficiency

Item	Yes	No	Details
External quantum efficiency (EQE) or incident photons to current efficiency (IPCE)	☒	☐	We provide EQE measurement in Fig. 3g and Supplementary Fig. 15.
A comparison between the integrated response under the standard reference spectrum and the response measure under the simulator	☒	☐	We compare the integrated Jsc with one from JV scan. The difference between the integrated Jsc from EQE and Jsc from JV scan is within 2% difference, which is within accuracy confidence of the measurements.
For tandem solar cells, the bias illumination and bias voltage used for each subcell	☐	☒	We didn't fabricate tandem solar cell.

5. Calibration

Item	Yes	No	Details
Light source and reference cell or sensor used for the characterization	☒	☐	See details in Method.
Confirmation that the reference cell was calibrated and certified	☒	☐	Our solar simulator was calibrated by Si reference cell.(certificated by Newport.)

Calculation of spectral mismatch between the reference cell and the devices under test	☒ Yes ☐ No	A spectral mismatch calculation was performed based on the spectral intensity of the solar simulator and 3 typical EQEs of our cells. This resulted in 2 mismatch factors of M = 0.9951 and 0.9968.

6. Mask/aperture

Size of the mask/aperture used during testing	☒ Yes ☐ No	An optical aperture mask (0.055 cm2) was used.
Variation of the measured short-circuit current density with the mask/aperture area	☐ Yes ☒ No	No significant variations was observed.

7. Performance certification

Identity of the independent certification laboratory that confirmed the photovoltaic performance	☐ Yes ☒ No	Our paper does not emphasize solar cell efficiency.
A copy of any certificate(s)<br>*Provide in Supplementary Information*	☐ Yes ☒ No	Our paper does not emphasize solar cell efficiency.

8. Statistics

Number of solar cells tested	☐ Yes ☒ No	Our paper does not emphasize solar cell efficiency.
Statistical analysis of the device performance	☐ Yes ☒ No	Our paper does not emphasize solar cell efficiency.

9. Long-term stability analysis

Type of analysis, bias conditions and environmental conditions<br>*For instance: illumination type, temperature, atmosphere humidity, encapsulation method, preconditioning temperature*	☐ Yes ☒ No	Stability was not related to our band extending concept.
